# Impact of Key Lifestyle Behaviors on Hypertension Control: Implications for Optimizing Patient Management

**DOI:** 10.3390/healthcare14010010

**Published:** 2025-12-19

**Authors:** Salihah Kashum, Ghareeb Bahari

**Affiliations:** 1 Jazan Health Cluster, Ministry of Health, Jazan 82611, Saudi Arabia; skashoom@moh.gov.sa; 2 Nursing Administration and Education Department, College of Nursing, King Saud University, Riyadh 11421, Saudi Arabia

**Keywords:** hypertension, lifestyle behaviors, self-confidence, blood pressure control

## Abstract

**Background/Objective**: Hypertension is a major global health concern and a leading cause of cardiovascular morbidity and mortality. Lifestyle behaviors, such as diet, physical activity, stress management, and self-confidence, markedly influence hypertension control. Exploring these behaviors can inform culturally relevant interventions for improving the prevention and management of hypertension and health outcomes of affected individuals. This study aimed to determine the effects of lifestyle behaviors, including dietary habits, physical activity, stress management, and self-confidence, on optimizing hypertension control among individuals in Saudi Arabia. **Methods**: A cross-sectional, descriptive–correlational design was used. Data were collected from 136 patients with hypertension attending primary healthcare centers in Saudi Arabia using validated scales for dietary habits, physical activity, perceived stress, and self-confidence, alongside blood pressure measurements. Data were analyzed using Statistical Package for the Social Sciences version 26. **Results**: The study revealed that most participants reported reasonably healthy dietary practices, low physical activity, and moderate stress and self-confidence. Significant sex differences (*p* < 0.05) were observed, with men and women reporting higher physical activity and stress, respectively. Education and age influenced dietary habits and self-confidence. Regression analysis identified age, education, and urban residence as predictors (*p* < 0.05) of blood pressure status, while stress, diet, and physical activity affected self-confidence and perceived stress levels. **Conclusions**: Hypertension management is influenced by interconnected lifestyle and psychosocial factors, and improving dietary habits, physical activity, stress management, and self-confidence is essential. Tailored interventions addressing demographic differences can enhance self-care behaviors and facilitate better hypertension control among Saudi individuals.

## 1. Introduction

Hypertension, a chronic medical condition marked by consistently high blood pressure, is a major risk factor for cardiovascular disease [1]. It has been reported to be one of the top causes of mortality worldwide [2], and it is estimated that one in every four men and one in every five women worldwide are affected by this major cause of early death [3]. Hypertension is a leading cause of morbidity and death in the Kingdom of Saudi Arabia. Research indicates that its prevalence in the country can reach up to 41.8% [4]. Further, almost 60% of individuals with hypertension in Saudi Arabia are undiagnosed [5].

The new hypertension categories outlined in the American College of Cardiology/American Heart Association guidelines for hypertension management are as follows: normal, systolic < 120 mmHg and diastolic < 80 mmHg; elevated, systolic 120–129 mmHg and diastolic < 80 mmHg; stage 1 hypertension, systolic 130–139 mmHg or diastolic 80–89 mmHg; and stage 2 hypertension, systolic ≥ 140 mmHg and diastolic ≥ 90 mmHg. These categories should be confirmed by two or more readings on at least two separate occasions, and individuals must be classified based on their highest systolic or diastolic blood pressure category. The pre-hypertension category is no longer used [6].

Effective lifestyle changes might delay the need for pharmacological therapy, contribute to improved blood pressure management, and minimize the amount of antihypertensive medication required [7]. Healthy lifestyle changes include reducing salt, sodium, and fat in the diet, modifying eating habits to include more vegetables and fruits, avoiding smoking, engaging in regular physical activity, maintaining a healthy body weight, and eliminating stress [8].

Recent studies have shown a strong connection between regular physical exercise and the management of hypertension [9,10], with regular exercise playing a key role in both the prevention and treatment of hypertension. Additionally, it is related to a decreased risk of cardiovascular disease and death, because it markedly improves cardiometabolic health and body composition, leading to better cardiac parasympathetic function by boosting the vagal tone. This results in lower resting heart rates and systolic blood pressure in adults with hypertension. To lower systolic blood pressure by up to 5 and 4 mmHg, respectively, aerobic and dynamic resistance training performed for 90 to 150 min per week has been anticipated to be effective.

Dietary behavior is a vital and continuous part of everyday life, involving internal, external, and conscious actions connected to eating [11]. Nutrition is markedly related to hypertension, and many studies have highlighted the importance of certain dietary patterns (such as the DASH [Dietary Approaches to Hypertension Control] and Mediterranean diet) in the management of hypertension [12]. Additionally, limiting salt intake could lower blood pressure and the risk of developing cardiovascular disease [7]. Further, several recommendations suggest that stress management should be used as an additional intervention for the treatment of hypertension [13]. It is especially advantageous for lowering the diastolic blood pressure [1].

One of the reasons why patients who are aware of their health care may not participate in collaborative decision-making with their doctors is that they lack confidence at the time. Having confidence, on the other hand, may also have some negative consequences [14]. Self-confidence is critical for maintaining emotional stability and managing stress effectively. It involves trusting in one’s own abilities, qualities, and judgment. This important trait can greatly influence many areas of life, such as personal relationships, career growth, and overall health [15]. Additionally, a greater degree of self-confidence was found to be positively associated with a greater degree of treatment adherence among patients with hypertension. It is feasible to improve the medication adherence of patients with hypertension by increasing their self-confidence [16].

Despite the currently available antihypertensive drugs being effective, there is a need for novel treatment options that are more successful in particular categories of individuals with hypertension, as well as for greater resources to manage hypertension. Systolic blood pressure can be reduced by 3.5 mmHg by maintaining healthy lifestyle variables, such as body mass index, food, smoking, salt excretion, and sedentary behavior. This is true regardless of whether an individual is genetically predisposed to hypertension [17].

Hypertension tops the list of risk factors for cardiovascular diseases that are highly incapacitating and fatal, thus resulting in high morbidity and mortality rates. However, optimal control of hypertension has remained a challenge despite the tremendous strides made in its medical treatment. Multiple lifestyle behaviors and self-confidence influence the control of hypertensive conditions since these factors are within one’s ability to change and have been shown to affect blood pressure regulation. However, little information is available on how these behaviors are related within the social and cultural norms of Saudi society. Therefore, this study aimed to determine the effects of major lifestyle factors, including dietary habits, physical activity, stress management, and self-confidence, on hypertension control among patients in Saudi Arabia. We believe this study has the potential to shed light on an important public health issue in Saudi Arabia, i.e., optimal management of hypertension, a highly prevalent condition.

### Theoretical Framework

The study used the self-efficacy theory [18], focusing on individuals’ belief in their capability to perform specific behaviors, influenced by four main sources of information: mastery experiences, vicarious experiences, verbal persuasion, and emotional arousal [19]. The first factor, *mastery experiences*, is when an individual’s self-belief is strengthened by successes and undermined by failures. The second factor is *vicarious experiences*, which involve individuals learning from observing others’ behaviors and their outcomes. The third factor is *social or verbal persuasion,* which is defined as encouragement or discouragement from others markedly shaping self-efficacy beliefs. Finally, *emotional arousal* plays a role in influencing self-efficacy.

The *mastery experiences* component enhances self-efficacy by increasing confidence when individuals succeed at tasks, while failures can lower that confidence. For instance, Dzerounian et al. [20] found that patients with higher self-efficacy were more likely to adhere to hypertension management behaviors, such as physical activity, stress management, and dietary habits. Emotions can also influence patients’ self-confidence in managing their chronic disease. Such factors were assumed to have positive relationships with patients’ self-efficacy and hypertension control.

## 2. Materials and Methods

### 2.1. Study Design and Setting

The study employed a cross-sectional, descriptive–correlational design whereby the relationships between the studied variables at a single point in time were explored. The study design enabled data collection from patients with hypertension to investigate their physical activity, dietary habits, stress management, and self-confidence levels. It offered a clear view of the study participants’ lifestyle behaviors and how these behaviors can influence hypertension control. The study was conducted in primary healthcare centers, because they are the initial points of care for regular visits for chronic condition treatment in Saudi Arabia.

### 2.2. Sampling Process

A convenient sampling method was used for data collection. This method helps gather data from a population that is easily accessible and readily available. It also offers several benefits, including reduced effort, low cost, simple access to a sample, and a lack of need for a comprehensive list of population elements while also providing valuable qualitative data. Participants aged 18 years or older with a clear diagnosis of hypertension by a healthcare professional who indicated their willingness to participate in the study and provided informed consent were included in the study.

Patients with cognitive impairment that could affect their ability to self-manage their condition were excluded, as they were unlikely to provide accurate or reliable responses. Pregnant women were also excluded because pregnancy naturally leads to changes in blood pressure, hormones, and metabolism that could influence hypertension and lifestyle habits and including them would have made it difficult to clearly determine how lifestyle factors alone affect blood pressure control. An electronic tool (https://www.danielsoper.com/statcalc/calculator.aspx?id=1, accessed on 15 August 2024) was used to estimate the recommended minimum sample size. This was completed with a statistical power of 0.8, an effect size of 0.15, an alpha level of 0.05, and 13 explanatory variables, which included 9 demographic variables and four main variables. The results indicated that a minimum sample size of 131 was required.

### 2.3. Instrumentation

A structured questionnaire survey including questions on participants’ age, sex, marital status, education level, income, residence, duration since being diagnosed with hypertension, presence of household members with hypertension, and number of household members living with the patient was administered to the participants. The Dietary Habits scale was used to determine the participants’ eating patterns in relation to hypertension control [21]. It consisted of eight items, and each question was scored on a 3-point Likert scale. The total score was calculated by summing the scores for all dietary questions, and a possible total score ranged from 0 to 16 points. A score between 12 and 16 points indicated optimal dietary habits highly recommended for hypertension control. This scale demonstrated a slightly low level of reliability (α = 0.61), suggesting that while the items were reasonably related, further refinement could improve coherence.

A physical activity questionnaire that focused on physical activity participation and evaluation of the frequency, duration, intensity, overall length, and types of activities in which a person engaged was used [22]. It included eight questions, and each question was scored on a 5-point Likert scale, with scores ranging from 1 to 5 points. The total score for physical activity was determined by summing the scores for all questions, and a possible total score ranged from 5 to 25 points. A score between 18 and 25 points indicated a high level of physical activity, reflecting that the participant was highly active—a behavior generally associated with better hypertension control. The physical activity scale achieved good reliability (α = 0.80) for internal consistency among items.

The perceived stress scale was used to measure the participants’ perceived stress levels during the previous month [23]. Each question was scored on a 5-point Likert scale, with the scores ranging from 0 to 4 points, where 0 points indicated the lowest frequency (“never”) and 4 points indicated the highest frequency (“very often”). The total score was calculated by summing the points for all 10 items on the scale, a possible total score ranged from 0 to 40 points. A score between 0 and 13 points indicated low stress levels. The perceived stress scale showed a reliability of 0.60, which is considered the lower limit of acceptability, suggesting that while the items were somewhat consistent in the measurement of perceived stress, there was likely room for improvement in item clarity, coverage, or homogeneity to enhance the scale’s reliability.

Participants’ confidence in managing their hypertension and daily health behaviors was also evaluated using the Self-efficacy for Managing Chronic Disease 6-item scale developed by the Self-Management Resource Center [https://shorturl.at/Re7et, accessed on 20 August 2024]. Each question was scored on a 10-point Likert scale, ranging from 1 to 10 points, with a score of 1 point representing the lowest confidence level (“not at all confident”) and a score of 10 points representing the highest confidence level (“totally confident”). The total score for the self-confidence assessment is calculated by summing the scores for all questions. A score between 45 and 60 points indicated high self-confidence, meaning the participant felt confident in managing their health and controlling hypertension. The self-efficacy scale had a high Cronbach’s alpha (α = 0.93), reflecting excellent internal agreement between items.

In addition to the survey questions, participants were asked to report their last blood pressure readings from a healthcare visit. If the systolic and diastolic pressure values were below 140 and 90 mmHg, respectively, they were considered to have controlled hypertension. Readings above these indicated uncontrolled hypertension.

### 2.4. Data Collection and Analysis

Data collection was conducted between December 2024 and March 2025 through an electronic survey platform using Google Forms. The Google Form allowed participants to easily complete the survey and was accessible only to the research team. This approach effectively maintained secure data storage, and the researchers continuously reviewed the data for accuracy and completeness throughout the data collection process. The survey link was shared with the participants through different digital platforms, such as email, social media, and messaging apps, to ensure ease of data collection. This allowed the participants to access and complete the questionnaire at their convenience, and enabled greater reach and rapid response collection.

The Statistical Package for the Social Sciences version 26 was used for data management and analysis. Before analysis, the data were carefully cleaned by checking for errors, inconsistencies, and missing values. The dataset had a very low rate of missing data, which ensured the accuracy and reliability of the analysis. Descriptive statistics were run to describe the demographic and main variables. Next, Pearson coefficient correlation test was run to determine associations between continuous variables. The independent-samples *t*-test was also used to compare the means of two separate groups for a single continuous dependent variable. One-way analysis of variance (ANOVA) was used to identify any significant differences among the means of three or more independent groups. In addition, multiple linear regression was employed when predicting the value of one variable based on the values of two or more other variables.

## 3. Results

### 3.1. Sociodemographic Characteristics

Table 1 presents the sociodemographic characteristics of the study participants. Most were aged 36–49 years (44.1%) and female (68.4%). Regarding clinical factors, 42.6% had been diagnosed with hypertension for less than 1 year, and 37.5% for more than 5 years. A large proportion (77.2%) reported household members with hypertension.

### 3.2. Statistical Analysis of the Main Variables

Table 2 presents the distribution of participants according to dietary habits, physical activity, perceived stress, and self-confidence. Most participants reported having reasonably healthy (45.6%) dietary habits. Physical activity levels were generally low. In terms of perceived stress, most participants (86.0%) experienced moderate stress. Regarding self-confidence, nearly equal proportions were classified as high (41.2%). Most of the participants were classified as hypertensive: 64 participants (47.06%) were in stage 1 hypertension.

The Pearson correlation analysis revealed several significant associations among the study variables. Total physical activity showed a small but significant negative correlation with perceived stress (r = −0.172, *p* < 0.05) and a moderate positive correlation with confidence (r = 0.281, *p* < 0.01). Perceived stress demonstrated a strong negative relationship with confidence (r = −0.484, *p* < 0.01). Confidence was also significantly and positively correlated with blood pressure (r = 0.182, *p* < 0.05). Additionally, systolic and diastolic blood pressure were strongly correlated with each other (r = 0.343, *p* < 0.01), reflecting their expected physiological relationship.

The mean differences in dietary habits, physical activity, perceived stress, and self-confidence across demographic factors, determined using the independent-samples *t*-test and one-way ANOVA, are presented in Table 3. Sex had a significant effect on physical activity (*p* < 0.001), with male patients reporting higher activity levels than those reported by female patients. Regarding perceived stress levels (*p* = 0.012), female patients reported higher stress scores than those reported by male patients. Overall, the findings highlight sex-related differences in physical activity and stress, while other demographic factors had minimal influence. One-way ANOVA revealed significant differences in dietary habits according to education (*p* = 0.023) and age (*p* = 0.002). Self-confidence differed significantly by education (*p* = 0.012), although participants living alone reported slightly higher self-confidence.

### 3.3. Analysis of Factors Affecting Blood Pressure

Table 4 shows the results of multiple linear regression analysis of predictors of blood pressure status among the participants. The model was significant (*p* = 0.034) and explained 18.4% to 25.4% of the variance (Cox & Snell R^2^ = 0.184). Age was a significant predictor (B = 0.046, *p* = 0.049), indicating that increasing age was associated with higher odds of abnormal blood pressure. Education was also an influencing factor; compared with university graduates, individuals with secondary education (B = –1.079, *p* = 0.042) and illiterate participants (B = –2.535, *p* = 0.011) were significantly less likely to maintain normal blood pressure. Other demographic and lifestyle factors were not significant predictors.

## 4. Discussion

This study was aimed at determining the effects of lifestyle behaviors, including dietary habits, physical activity, and stress management, and self-confidence, on the optimization of hypertension control in Saudi Arabia. The study results indicated that the dietary practices of the majority of its participants were reasonably healthy (46.6%); 37.5% and 10.3% of the participants reported good and poor dietary practices, respectively. The percentage of those with excellent dietary practices was only 2.9%, thus indicating a huge gap between recommended guidelines and actual practice. The study findings reaffirm those of earlier studies that showed that dietary behaviors continue to be the major impediment in hypertension control. Studies revealed that dietary approaches, particularly adherence to the DASH and Mediterranean diets, significantly reduced systolic as well as diastolic blood pressure [24,25]; however, adherence was quite poor among most populations. Structured interventions involving the DASH diet at primary care levels improved cardiometabolic parameters; these findings supported reported previously [25].

The study showed that most of the participants had low physical activity. Approximately 41.2% stated that they did not engage in regular structured exercise, while 4.4% reported being involved in a high level of physical activity. Furthermore, 35.3% of the participants reported engaging in low physical activity, while approximately 19.1% reported moderate levels of activity. This indicates physical exercise among the participants fell short of international recommendations, which call for at least 150 min of moderate-intensity or 75 min of vigorous-intensity activity per week to ensure optimal blood pressure levels. Evidence from structured interventions showed that physical activity can greatly lower blood pressure. For instance, Marin-Couture et al. [26] showed that supervised exercise protocols in Canadian primary-care patients with stage 1 hypertension greatly improved blood pressure and cardiometabolic outcomes.

In addition, systematic reviews and meta-analyses showed different clinically meaningful reductions in blood pressure with leisure-time and structured exercise. For example, Monfared et al. [27] shared good reductions (−7.7/−3.6 mmHg) across randomized controlled trials in low- and middle-income countries. When compared with these findings, the low levels of activity in the current study may explain why many participants struggle to control their hypertension even with medical care. Cultural attitudes and inadequate provisions for safe exercise facilities, socioeconomic constraints, and other competing priorities, seemingly in that order of importance in low- and middle-income settings, explain barriers to the uptake of physical activity interventions [28].

The present study found significant sex-related differences in physical activity and perceived stress, with male participants engaging in higher activity levels and female participants reporting higher stress scores. These findings align with previous research where Rachmawati et al. [28] noted that physical activity strongly affects blood pressure control, and sex-related differences in lifestyle behaviors may partially explain these outcomes. Elevated stress among female individuals has also been consistently reported, with psychosocial stressors such as marital and occupational roles contributing to heightened vulnerability [13,29]. Such results reinforce the need for sex-sensitive interventions that address both behavioral and psychosocial risk factors.

Age and education appeared to be significant predictors of dietary habits and self-confidence. Illiterate participants and those aged 50 years or older reported higher dietary habit scores, suggesting that older adults and individuals with lower literacy levels may rely more on traditional or culturally established eating patterns that align closely with healthy behaviors. Participants with a university education demonstrated higher self-confidence, which may reflect greater health literacy, better access to health information, and a stronger ability to understand and implement hypertension management strategies. These findings align with evidence [30], where researchers showed that education was a key determinant of positive health behaviors, and by Choi and Kim [31], who showed age-specific patterns in hypertension control among older adults. The paradoxical finding of better dietary scores among older and less educated participants may be explained by traditional dietary practices (e.g., low consumption of processed food) among older adults, consistent with cultural dietary considerations described by Miezah and Hayman [32]. Higher self-confidence among university graduates supports the self-efficacy framework [18,33], emphasizing the role of knowledge and *mastery experiences* in shaping health behaviors.

Participants without household members with hypertension exhibited a near-significant advantage in terms of dietary habits. Although the association was not statistically significant, it suggests that households without chronic illness may have fewer dietary restrictions or stressors related to caregiving. Previous evidence highlighted the role of family involvement in shaping adherence [34], but also revealed gaps between family support and lifestyle adherence. This suggests that family health history may help and limit lifestyle choices depending on family dynamics and awareness.

The regression model in this study was statistically significant, indicating that age, education, and residence were the strongest predictors of blood pressure status, yet other demographic and lifestyle factors showed limited predictive values. The significance of residence further emphasizes how environmental and contextual factors may influence blood pressure outcomes. These findings suggest that differences in lifestyle and access to healthcare services across residential settings may contribute to variations in blood pressure status. Thus, further research is warranted to comprehensively explore these contextual influences and to examine how residential areas interact with effective hypertension management. The finding that older age was associated with abnormal blood pressure is consistent with previous evidence provided by Liew et al. [34], who reported age as one of the most consistent sociodemographic correlates of hypertension prevalence and control. Similarly, Choi and Kim [31] found that advancing age in Korea was linked to poor blood pressure control, although they also observed protective associations in specific older subgroups. The results of the present study confirm the established biological link between vascular stiffening, endothelial dysfunction, and increased arterial resistance with age, making age a non-modifiable but critical predictor of hypertension outcomes.

Education level was an influential determinant of hypertension control: both secondary school and illiterate participants were significantly less likely to maintain normal blood pressure compared to university graduates. These results support previous findings that higher education correlated with better self-care and medication adherence [35,36]. Education may improve health literacy, enabling patients to understand treatment regimens, follow dietary guidance, and engage in self-monitoring. Conversely, low education levels may limit access to health knowledge and diminish confidence in managing chronic diseases. These findings reinforce the central role of patient education in hypertension management, aligning with Bandura’s self-efficacy theory, which assumes that knowledge and *mastery experiences* build stronger self-belief in health behaviors.

Urban residents in this study were significantly less likely to maintain normal blood pressure compared to rural participants. This result aligns with evidence [37] in China, who found that dietary transitions toward processed and high-sodium foods in urban settings contributed to poor hypertension control. It also reflects lifestyle patterns such as higher stress levels, sedentary behaviors, and environmental pollution in urban areas. Conversely, rural diets often emphasize traditional foods with lower salt content, which may offer a protective effect [32]. This highlights the need for specific interventions that address the unique risks of urban environments, such as reducing dietary sodium and promoting workplace-based physical activity and stress management.

Sex, marital status, income, household size, and household hypertension history were not significant predictors in the final model. While these factors have been highlighted in other research, their lack of significance in this study may reflect sample characteristics or cultural homogeneity within the study population. Likewise, lifestyle variables such as dietary habits, physical activity, stress, and self-confidence did not reach statistical significance despite their strong associations with hypertension management in the literature [13,36,37].

### 4.1. Limitations of the Study

Several limitations should be noted. The present study used a self-reported questionnaire, which may introduce bias or inaccuracy and limits the researchers’ ability to verify respondent identity or control the conditions under which the questionnaire was completed. Since it was a cross-sectional study, it captured the association at one time, and therefore, causality between lifestyle behaviors and blood pressure outcomes cannot be made. Additionally, the convenience sampling strategy and relatively small sample size limit the generalizability of the findings to other populations or cultural contexts. Potential cultural and sex-related barriers that may influence lifestyle behaviors also represent limitations warranting further investigation. Other unmeasured factors that may influence the control of blood pressure, such as taking medication correctly or genetic predisposition, were not included in this study.

### 4.2. Study Implications

Based on the findings of this study, future research should consider adopting longitudinal or experimental designs to better establish causal relationships between lifestyle behaviors and blood pressure outcomes. Expanding the sample size and including participants from diverse geographic and cultural contexts would improve the generalizability of the results. Additionally, integrating factors such as medication adherence, genetic predispositions, and healthcare access could provide a more comprehensive understanding of hypertension control. Finally, interventions targeting lifestyle modifications should be tailored to demographic patterns identified in this study to enhance their effectiveness in population-specific settings.

Community health nurses are key players in the promotion of lifestyle modification for patients with hypertension. There is a pressing need for these nurses not to be knowledge providers but rather to help patients translate awareness into concrete, sustainable behaviors. Our study findings suggest that counseling about lifestyles and patient self-efficacy should be made part of the regular clinical practice. This can be facilitated by nurse managers through the provision of adequate training, resources, and time allocation for staff to involve patients in health promotion activities.

Furthermore, community engagement projects and simulations that would practically strengthen the ability to effectively support patients with regard to diet and exercise as well as stress management are welcomed. Additionally, there are gaps between lifestyle behaviors and hypertension outcomes that will define multiple tracks of future research. Longitudinal and interventional studies are, therefore, proposed to ascertain the causal relationship between lifestyle modification, self-efficacy, and blood pressure control.

## 5. Conclusions

Our results indicated that most of the respondents had reasonably healthy dietary practices, low physical activity, moderate stress, and moderate self-confidence, i.e., gaps in adherence to the lifestyle required for hypertension control. The study found differences between the sexes, with men reporting higher levels of physical activity and women reporting higher stress. Education and age were determinants of dietary behaviors and self-confidence. Regression analysis also found age, education, and urban residence to be strong predictors of blood pressure status. The study findings indicate that hypertension management is not only dependent on clinical treatment alone but also on psychosocial and lifestyle factors.

## Figures and Tables

**Table 1:** Demographic characteristics (N = 136).

Variables	Frequency (%)
Age groups	
≤35 years	29 (21.3)
36–49 years	60 (44.1)
≥50 years	47 (34.6)
Sex	
Male	43 (31.6)
Female	93 (68.4)
Marital status	
Married	112 (82.4)
Not married	24 (17.6)
Education	
University	77 (56.6)
Secondary school or less	45 (33.1)
Illiterate	14 (10.3)
Income	
≤7000 SAR	64 (47.1)
>7000 SAR	72 (52.9)
Residence	
Rural area	46 (33.8)
Urban area	90 (66.2)
Time since diagnosis	
<1 year	58 (42.6)
1 to 5 years	27 (19.9)
>5 years	51 (37.5)
Household members with hypertension	
Yes	105 (77.2)
No	31 (22.8)
Household members living with patient	
Living alone	10 (7.4)
1 to 5 members	71 (52.2)
>5 members	55 (40.4)

**Table 2:** Participants’ distribution according to the main variables.

Variables	Categories	Frequency (%)
Dietary habits	Poor	14 (10.3)
	Reasonably health	62 (45.6)
	Good	51 (37.5)
	Excellent	4 (2.9)
Physical activity	Very low	56 (41.2)
	Low	48 (35.3)
	Moderate	26 (19.1)
	High	6 (4.4)
Perceived stress	Low	10 (7.4)
	Moderate	117 (86.0)
	High	9 (6.6)
Self-confidence	Low	25 (18.4)
	Moderate	55 (40.4)
	High	56 (41.2)
Blood pressure reading	Normal	12 (8.82)
	Elevated	18 (13.24)
	Hypertension stage 1	64 (47.06)
	Hypertension stage 2	38 (27.94)
	Hypertensive crisis	4 (2.94)

**Table 3:** Mean differences in the main variables across some demographic factors.

Variable	Binary Categories	N	Dietary Habits M (SD)	*p*-Value	Physical Activity M (SD)	*p*-Value	Stress M (SD)	*p*-Value	Self-Confidence M (SD)	*p*-Value
Sex	Male	43	16.28 (2.65)	0.157	2.34 (0.90)	0.000 *	19.02 (5.12)	0.012 *	6.67 (2.01)	0.201
	Female	93	15.54 (2.90)		1.73 (0.66)		21.12 (4.13)		6.12 (2.47)	
Marital status	Married	112	15.74 (2.77)	0.784	1.92 (0.79)	0.814	20.33 (4.40)	0.489	6.11 (2.33)	0.054
	Not married	24	15.92 (3.17)		1.96 (0.82)		21.04 (5.27)		7.13 (2.28)	
Income	≤7000 SR	64	15.88 (3.03)	0.891	1.92 (0.78)	0.948	20.08 (4.41)	0.363	6.32 (2.36)	0.903
	>7000 SR	72	15.68 (2.67)		1.93 (0.81)		20.79 (4.68)		6.27 (2.34)	
Residence	Rural	46	16.22 (2.82)	0.192	2.00 (0.88)	0.398	21.07 (4.21)	0.226	5.88 (2.16)	0.147
	Urban	90	15.54 (2.83)		1.88 (0.75)		20.14 (4.70)		6.50 (2.42)	
Time since diagnosis	<5 years	58	15.47 (2.77)	0.220	1.97 (0.80)	0.443	20.66 (4.51)	0.414	6.66 (2.36)	0.387
	≥5 years	27	14.63 (3.18)		2.11 (0.84)		19.70 (5.86)		6.18 (2.33)	
Household member with hypertension	Yes	105	15.51 (2.85)	0.051	1.91 (0.77)	0.687	20.77 (3.87)	0.137	6.22 (2.35)	0.508
	No	31	16.65 (2.67)		1.97 (0.90)		19.39 (6.30)		6.54 (2.35)	
Education	University	77	15.21 (3.05)	0.023 *	2.01 (0.84)	0.263	20.35 (4.65)	0.299	6.56 (2.53)	0.012 *
	Secondary school or less	45	16.38 (2.27)		1.85 (0.77)		20.09 (4.67)		6.36 (2.07)	
	Illiterate	14	16.93 (2.64)		1.67 (0.56)		22.21 (3.26)		4.56 (1.14)	
Age groups	≤35 years	29	14.93 (3.15)	0.002 *	1.96 (0.76)	0.463	20.45 (4.73)	0.429	6.79 (2.47)	0.408
	36–49 years	60	15.28 (2.79)		2.00 (0.88)		20.97 (4.06)		6.08 (2.48)	
	≥50 years	47	16.91 (2.35)		1.81 (0.69)		19.81 (5.02)		6.26 (2.06)	
Household members	Living alone	10	14.10 (3.14)	0.096	1.86 (0.81)	0.403	18.00 (5.08)	0.185	7.80 (2.33)	0.103
	1 to 5 members	71	15.69 (2.70)		2.01 (0.81)		20.48 (5.31)		6.22 (2.52)	
	>5 members	55	16.18 (2.89)		1.82 (0.76)		20.87 (3.10)		6.12 (2.03)	

* *p*-value < 0.05.

**Table 4:** Multiple regression analysis of the factors affecting blood pressure.

Variables	Unstandardized Coefficients (B)		Standardized Coefficients	Sig.	Model Summary	
	B	Std. Error	Exp(B)		R^2^	*p*-Value
Age	0.046	0.023	1.047	0.049 *	0.184	0.034 *
Sex	0.508	0.580	1.662	0.381		
Marital Status	−0.352	0.589	0.703	0.550		
Education (secondary vs. university)	−1.079	0.531	0.340	0.042 *		
Education (none vs. university)	−2.535	1.000	0.079	0.011 *		
Income	−0.684	0.491	0.505	0.163		
Residence	−1.526	0.522	0.217	0.003 *		
Duration	0.439	0.598	1.551	0.463		
Household member with hypertension	−0.135	0.539	0.874	0.802		
Household size	−1.286	1.161	0.276	0.268		
Total dietary habits	−0.069	0.080	0.934	0.392		
Physical activity	0.177	0.318	1.193	0.579		
Stress	0.060	0.055	1.061	0.277		
Self-confidence	0.139	0.107	1.149	0.193		
Constant	0.310	2.629	1.363	0.906		

* *p*-value < 0.05. Note: Age was measured as a continuous variable, and some variables were collapsed into binary categories.

## Data Availability

The datasets generated and analyzed during the current study are not publicly available owing to privacy and ethical restrictions but are available from the corresponding author upon reasonable request.

## Abbreviations

The following abbreviations are used in this manuscript:

DASH	Dietary Approaches to Hypertension Control
ANOVA	Analysis of variance
